# Clinical Characteristics and Mortality-Associated Factors in Trauma Patients Undergoing Permanent Versus Temporary Tracheostomy

**DOI:** 10.3390/clinpract15010012

**Published:** 2025-01-04

**Authors:** Ahmad K. Alnemare

**Affiliations:** Department of Otolaryngology, Faculty of Medicine, Majmaah University, Al-Majmaah 11952, Saudi Arabia; a.alnemare@mu.edu.sa

**Keywords:** tracheostomy, mortality, national trauma data bank, outcomes, risk factors

## Abstract

**Objective:** This study evaluated the characteristics, outcomes, and mortality-associated factors in patients who underwent tracheostomy after traumatic injury to optimize clinical decision-making and patient care in critical trauma settings. **Materials and Methods:** A retrospective cohort analysis was conducted using the National Trauma Data Bank (NTDB) records from 2013 to 2016. This study included 41,630 adult trauma patients who underwent tracheostomy procedures. Data analysis included descriptive statistics, univariate comparisons, and multivariate logistic regression analyses. The study protocol adhered to STROBE guidelines for observational studies. **Results:** Analysis of the total cohort revealed that patients with tracheostomy demonstrated high rates of severe injuries (75.2%) and a notable comorbidity burden, including cardiovascular disorders (4.0%) and blood disorders (5.8%). Multivariate analysis revealed that mortality risk was independently associated with advanced age (OR 1.018, 95% CI 1.016–1.021), higher injury severity scores (OR 1.004, CI 1.002–1.007), female sex (OR 1.187, CI 1.078–1.308), and cardiovascular surgical intervention (OR 1.487, CI 1.350–1.638). Among the study population, 7.6% underwent permanent tracheostomy procedures, with these patients showing some distinct clinical characteristics in terms of injury severity and comorbidity profiles. **Conclusions:** This comprehensive analysis demonstrates the complex clinical characteristics and mortality-associated factors in trauma patients requiring tracheostomy. Key factors influencing survival outcomes include age, injury severity, sex, and cardiovascular surgical intervention. These findings provide valuable insights for clinical decision-making and risk assessment in trauma patients requiring tracheostomy. The observed differences between permanent and temporary tracheostomy patients warrant further investigation with more detailed timing and indication data.

## 1. Introduction

Tracheostomy is a common surgical procedure performed in critically ill patients who require prolonged mechanical ventilation or airway protection when there is obstruction of airflow at or above the level of the larynx [1,2,3]. In traumatic injuries, tracheostomy may be necessary to manage acute respiratory failure, facilitate weaning from mechanical ventilation, or protect the airway in patients with severe head or neck injuries [4,5,6]. It can be performed as a permanent or temporary measure depending on the patient’s specific needs and anticipated duration of mechanical ventilation.

Both temporary and permanent tracheostomies have specific indications, complications, and care requirements [7,8]. Temporary tracheostomies are crucial in acute, life-threatening situations, and are designed to be reversible once the underlying condition improves [9]. Permanent tracheostomies, while less common, offer a long-term solution for patients with chronic airway obstruction or neurological conditions, providing a tube-free alternative that can significantly improve their quality of life [10]. Regardless of the type, diligent patient management and monitoring are essential for minimizing risks and complications [11].

In trauma patients, tracheostomy is a key intervention, particularly in cases of severe injury, and its timing and necessity vary based on the type of trauma and patient demographics [3,12,13]. A National Trauma Data Bank (NTDB) study focusing on pediatric patients showed that tracheostomy rates were significantly higher in children treated at adult or combined adult/pediatric trauma centers than in those treated at pediatric centers, suggesting differences in clinical practices or patient populations across these settings [14]. This is echoed in the broader trauma population, where early tracheostomy, defined as within 7 days of injury, is associated with reduced in-hospital complications, shorter critical care stays, and less time on mechanical ventilation for patients with traumatic cervical spinal cord injury (SCI) [15]. A systematic review supports the benefits of early tracheostomy in patients with acute traumatic SCI, highlighting reductions in mechanical ventilation duration, ICU stay, and hospital stay, although it does not conclusively affect short-term mortality [16]. Similarly, early tracheostomy has been shown to reduce the ventilation duration and ICU stay in expediting recovery [17]. In patients with severe chest trauma, certain risk factors such as prehospital endotracheal intubation and diagnosis of pneumonia during ICU stay increase the likelihood of tracheostomy [18]. Conversely, for patients undergoing anterior cervical discectomy and fusion, tracheostomy did not increase the risk of infection or nonunion despite the proximity of the surgical sites. Factors influencing tracheostomy in traumatic C3–C5 SCI include lower Glasgow Coma Scale scores and higher initial rapid shallow breathing index [14]. Complications associated with tracheostomy procedures, such as peristomal granulation tissue, vary with the surgical approach and initial tracheostomy tube size, indicating the importance of technique and equipment choice [19]. In patients with rib fractures, tracheostomy requirements are influenced by injury severity, with more severely injured patients and those with more extensive rib fractures being more likely to undergo the procedure [20]. All of these studies underscore the complexity of decision-making regarding tracheostomy in trauma patients, highlighting the influence of injury type, severity, patient age, and care setting on outcomes.

Despite the widespread use of tracheostomy in trauma patients, there are limited data on the characteristics, outcomes, and factors associated with mortality in this patient population [21]. Understanding the clinical profile, risk factors, and outcomes of trauma patients requiring tracheostomy is crucial for guiding clinical decision-making and optimizing management strategies [22]. This study aims to address the knowledge gap regarding tracheostomy use in trauma patients by analyzing a large, representative sample from the NTDB. We examined various factors associated with trauma severity, preexisting health conditions, management approaches, and clinical outcomes, while also exploring patterns related to tracheostomy duration as a secondary objective.

## 2. Methodology

### 2.1. Study Design

This study employed a retrospective cohort design using data from the NTDB, a large representative database of patients with trauma in the United States. The NTDB contains detailed information on patient demographics, injury characteristics, clinical interventions, and outcomes from participating trauma centers across the country.

### 2.2. Patient Population

This study included adult patients (aged 18 years or older) who underwent tracheostomy after traumatic injury between 1 January 2013, and 31 December 2016. Patients were identified using the International Classification of Diseases, 9th Revision, Clinical Modification (ICD-9-CM) procedure codes for tracheostomy (31.1, 31.2, 31.21, and 31.29). Patients were excluded if they had incomplete data on key variables such as age, injury severity score (ISS), or mortality.

### 2.3. Data Collection

Data were extracted from the NTDB using standardized data fields and definitions. The following variables were collected for each patient: age, sex, race, mechanism of injury, intent of injury, ISS, Glasgow Coma Scale (GCS) score, systolic blood pressure (SBP), type of tracheostomy (permanent or temporary), comorbidities, complications, length of hospital stay (LOS), intensive care unit (ICU) stay, and in-hospital mortality.

### 2.4. Definitions

Tracheostomy was defined as a surgical airway procedure creating a direct opening into the trachea for airway management [23]. In the clinical context, the duration of tracheostomy may vary based on patient needs and clinical circumstances, with some patients requiring extended or indefinite airway support while others may proceed to eventual decannulation [7,8,9]. The decision regarding tracheostomy duration is typically made based on multiple clinical factors, including the severity of the underlying condition, recovery potential, and overall patient prognosis.

For this study, tracheostomy classification was based on ICD-9-CM procedure codes documented at the time of surgery, rather than the subsequent clinical outcomes. Temporary tracheostomy (ICD-9-CM code 31.1) represented procedures specifically intended for temporary airway management, while permanent tracheostomy procedures encompassed non-specific permanent (31.2), mediastinal (31.21), and other permanent tracheostomy procedures (31.29).

Severe injury was defined as an Injury Severity Score (ISS) > 15, hemodynamic instability as systolic blood pressure (SBP) < 90 mmHg, and severe neurological impairment as Glasgow Coma Scale (GCS) ≤ 8.

### 2.5. Statistical Analysis

Descriptive statistics were used to summarize the patient characteristics, clinical variables, and outcomes. Continuous variables are reported as medians with interquartile ranges (IQR), while categorical variables are reported as frequencies and percentages. Comparisons between patients undergoing tracheostomies were performed using the Wilcoxon rank-sum test for continuous variables and the chi-square test or Fisher’s exact test for categorical variables, as appropriate. Logistic regression analysis was used to identify the factors associated with in-hospital mortality. The multivariate logistic regression model included variables with a *p*-value < 0.1 in the univariate analysis. Odds ratios (OR) with 95% confidence intervals (CI) were calculated for each variable in the final model. Model performance was assessed using the area under the receiver operating characteristic (ROC) curve. All statistical analyses were performed using STATA version 15.0 (StataCorp, College Station, TX, USA). Statistical significance was defined as a two-sided *p*-value < 0.05.

### 2.6. Ethical Considerations

This study was deemed exempt from institutional review board (IRB) approval due to the use of a de-identified, publicly available database. NTDB maintains strict data privacy and security protocols to ensure patient confidentiality.

## 3. Results

Table 1 presents a comparison of demographics and clinical characteristics between patients who underwent permanent tracheostomy (*N* = 3170) and those who underwent temporary tracheostomy (*N* = 38,460). The median age was similar in both groups, with 48.0 years (Q1, Q3:29.0, 63.0) in the permanent tracheostomy group and 47.0 years (Q1, Q3:29.0, 62.0) in the temporary tracheostomy group (*p* = 0.763). The proportion of patients aged ≥ 65 years was also comparable between the two groups (22.6% vs. 21.7%, *p* = 0.231). There was no significant difference in the sex distribution between the permanent and temporary tracheostomy groups (22.9% vs. 23.8% female, *p* = 0.548). The distribution of race differed slightly between the two groups (*p* = 0.045), with a higher proportion of white patients in both the permanent (67.9%) and temporary (67.9%) tracheostomy groups, followed by black patients (17.3% and 16.0%, respectively) and patients of other races (14.8% and 16.1%, respectively). The mechanism of injury was similar between the permanent and temporary tracheostomy groups (*p* = 0.430). Motor vehicle trauma (MVT) was the most common mechanism (47.4% and 48.4%, respectively), followed by falls (23.4% and 22.1%, respectively), firearm injuries (9.6% and 9.7%, respectively), and other mechanisms (11.8% and 11.4%, respectively). The intent to injure was also comparable between the two groups (*p* = 0.364), with unintentional injuries being the most prevalent (84.1% and 83.6%, respectively). Regarding comorbidities, patients who underwent permanent tracheostomy had a higher prevalence of cardiovascular health issues (4.7% vs. 3.9%, *p* = 0.042) and blood disorders (6.7% vs. 5.7%, *p* = 0.022) than those who underwent temporary tracheostomy. The prevalence of major psychiatric illness or dementia (10.6% vs. 10.6%, *p* = 0.972) and chronic health conditions (13.1% vs. 12.9%, *p* = 0.772) was similar between the two groups.

Table 2 presents a comparative analysis of the clinical metrics and demographic characteristics according to tracheostomy type. The median total GCS score was 6.0 (Q1, Q3:3.0, 14.0) in both the permanent and temporary tracheostomy groups (*p* = 0.686). The median SBP was also similar between the two groups, with 130.0 mmHg (Q1, Q3:104.0, 152.0) for the permanent tracheostomy group and 130.0 mmHg (Q1, Q3:108.0, 151.0) for the temporary tracheostomy group (*p* = 0.403). The proportion of patients with severe injury (ISS > 15) was significantly higher in the permanent tracheostomy group (78.2% vs. 74.9%, *p* < 0.001). However, there was no significant difference in the proportion of hemodynamically unstable (SBP < 90 mmHg) (25.0% vs. 25.8%, *p* = 0.329) or comatose patients (GCS total score ≤ 8) (56.8% vs. 56.1%, *p* = 0.467) between the two groups. The distribution of hospital types differed significantly between the two groups (*p* < 0.001), with a higher proportion of patients in the permanent tracheostomy group being treated at for-profit hospitals (16.4% vs. 11.0%) and a lower proportion of non-profit hospitals (83.6% vs. 89.0%) than in the temporary tracheostomy group.

Table 3 presents a comparison of the clinical outcomes and complications according to tracheostomy type. The median total ICU length of stay was significantly longer in the permanent tracheostomy group (19.0 days; Q1, Q3:13.0, 27.0) compared than in the temporary tracheostomy group (18.0 days; Q1, Q3:12.0, 26.0) (*p* < 0.001). The proportion of patients who underwent nose, mouth, and pharynx surgeries was significantly lower in the permanent tracheostomy group (10.0% vs. 12.2%, *p* < 0.001). In contrast, the proportion of patients who underwent surgeries of the cardiovascular system was significantly higher in the permanent tracheostomy group (74.2% vs. 71.2%, *p* < 0.001). There was no significant difference in the proportion of patients who underwent digestive system operations between the two groups (74.0% vs. 72.7%, *p* = 0.118). However, no significant difference was observed in the proportion of patients with surgical complications between the two groups (4.4% vs. 5.1%, *p* = 0.084). The mortality rates were similar between the permanent and temporary tracheostomy groups (4.8% vs. 4.8%, *p* = 0.995).

Table 4 presents the demographics and pre-existing conditions associated with mortality outcomes. Patients who died were significantly older (median age: 57.0 years; Q1, Q3:40.0, 70.0) than those who survived (median age: 47.0, Q1, Q3:29.0, 61.0) (*p* < 0.001). The proportion of patients aged ≥ 65 years was also significantly higher among those who died (35.7% vs. 20.7%, *p* < 0.001). There was a slightly lower proportion of females among those who died (22.0% vs. 23.8%, *p* = 0.048). The distribution of race categories was similar between the two groups (*p* = 0.237). However, the distribution of grouped mechanism categories differed significantly between those who died and those who survived (*p* < 0.001), with a higher proportion of falls (27.5% vs. 21.8%) and a lower proportion of MVT (41.8% vs. 48.8%) among those who died. The distribution of intent also differed significantly between the two groups (*p* < 0.001), with a higher proportion of self-inflicted injuries (6.1% vs. 4.5%) and a lower proportion of assault-related injuries (8.4% vs. 10.9%) among those who died. The distribution of trauma types varied significantly between the two groups (*p* < 0.001), with a higher proportion of burn injuries (5.1% vs. 1.5%) and a lower proportion of blunt injuries (79.1% vs. 83.3%) among those who died. Patients who died had a significantly lower prevalence of substance abuse (8.4% vs. 10.7%, *p* < 0.001) and major psychiatric illnesses or dementia (9.3% vs. 10.7%, *p* = 0.015) than those who survived. However, they had a significantly higher prevalence of cardiovascular health issues (7.6% vs. 3.7%, *p* < 0.001), chronic health conditions (19.4% vs. 12.5%, *p* < 0.001), blood disorders (9.7% vs. 5.4%, *p* < 0.001), and other major health conditions (4.1% vs. 2.2%, *p*< 0.001) than those who survived.

Table 5 presents a comparison of clinical and demographic factors based on mortality outcomes. The median total Glasgow Coma Scale score was slightly lower among the patients who died (5.0; Q1, Q3:3.0, 14.0) than among those who survived (6.0; Q1, Q3:3.0, 14.0) (*p* = 0.004). The median emergency department systolic BP was also significantly lower among patients who died (124.0 mmHg; Q1, Q3:100.0, 150.0) than among those who survived (130.0 mmHg; Q1, Q3:108.0, 151.0) (*p* < 0.001).

The proportion of severely injured patients was significantly higher among those who died (77.3% vs. 75.0%, *p* = 0.005). The proportion of hemodynamically unstable patients (31.1% vs. 25.3%, *p* < 0.001) and comatose patients (58.7% vs. 56.0%, *p* = 0.003) was also significantly higher among those who died. There was no significant difference in the proportion of patients who underwent interfacility transfer between the two groups (25.8% vs. 24.9%, *p* = 0.379).

Table 6 presents the clinical outcomes and procedure indicators according to mortality status. The median total ICU length of stay was shorter among patients who died (17.0 days; Q1, Q3:9.0, 27.0) than among those who survived (18.0 days; Q1, Q3:13.0, 26.0) (*p* < 0.001). The distribution of tracheostomy types (permanent or temporary) was similar between the two groups (*p* = 0.929).

The proportion of patients who underwent surgery on the nose, mouth, and pharynx was significantly lower among those who died (6.4% vs. 12.5%, *p* < 0.001). In contrast, the proportion of patients who underwent surgeries of the cardiovascular system was significantly higher among those who died (77.9% vs. 71.0%, *p* < 0.001). The proportion of patients who underwent digestive system surgeries was significantly lower among those who died (64.5% vs. 73.5%, *p* < 0.001).

Table 7 presents the results of the logistic regression analysis examining the factors associated with mortality (DUC) in patients undergoing tracheostomy after traumatic injury. The analysis revealed that age (odds ratio [OR], 1.018; 95% confidence interval [CI], 1.016–1.021; *p* < 0.001), ISS (OR, 1.004; 95% CI, 1.002–1.007; *p* < 0.001), and cardiovascular surgery (OR: 1.487; 95% CI: 1.350–1.638; *p* < 0.001) were significantly associated with increased odds of mortality. In contrast, higher SBP (OR, 0.999; 95% CI, 0.998–1.000; *p* = 0.021) and higher GCS scores (OR, 0.987; 95% CI, 0.979–0.994; *p* < 0.001) were associated with decreased odds of mortality. Tracheostomy type (permanent versus temporary) was not significantly associated with mortality (OR, 1.004; 95% CI, 0.864–1.167; *p* = 0.958). Other mechanisms of injury, including falls, firearm injuries, MVT, and others, were not significantly associated with mortality. The area under the ROC curve for this logistic regression model was 0.6413, indicating moderate predictive ability of the model for mortality outcomes.

## 4. Discussion

Comparative analysis of patient characteristics revealed distinct clinical patterns between tracheostomy groups. Patients discharged with ongoing tracheostomy requirements demonstrated higher rates of cardiovascular comorbidities and hematologic disorders compared to those who underwent decannulation or had planned decannulation. While demographic characteristics, including median age, sex distribution, and injury mechanisms, showed no significant differences between groups, clinical severity markers varied substantially. Specifically, patients requiring long-term tracheostomy care exhibited higher injury severity scores and were more frequently treated at for-profit institutions. These patients also experienced prolonged intensive care unit stays and underwent more frequent cardiovascular surgical interventions.

These differences suggest that patients requiring permanent tracheostomy may have more complex medical conditions and severe injuries, which could influence their outcomes and management strategies [10,23,24]. Compared to other studies, the findings of this study are consistent with previous research that has shown similar demographics and clinical characteristics between patients undergoing permanent and temporary tracheostomies. For example, Cheung and Napolitano (2014) found no significant differences in age, sex, or mechanism of injury between patients undergoing different types of tracheostomies [25]. Predictors of tracheostomy in trauma patients include patient-specific factors (such as age and comorbidities), diagnostic elements (type of injury and injury severity score), and intervention-related factors (such as craniotomy or laparotomy) [26]. However, the current study’s findings regarding the higher prevalence of cardiovascular health issues and blood disorders in the permanent tracheostomy group are in contrast with some previous studies. Kojicic et al. (2011) found no significant differences in comorbidities between patients undergoing different types of tracheostomy [27].

Interestingly, tracheostomy type (permanent vs. temporary) was not significantly associated with mortality. These findings are in agreement with a recent systematic review and meta-analysis of tracheostomy timing and its association with reported all-cause overall mortality, reporting no statistically significant association between tracheotomy timing and mortality (β = −0.3, 95% CI = −2.3 to 1.8) [28]. Premraj et al. focused on critically ill stroke patients and did not compare the outcomes of temporary and permanent tracheostomies. Our findings suggest that the decision to perform permanent or temporary tracheostomy may depend on factors beyond the risk of mortality. These factors include the expected duration of mechanical ventilation, underlying medical conditions, and potential need for long-term airway management [29,30].

Several factors are associated with mortality in patients undergoing tracheostomy after a traumatic injury [31,32]. Older age, higher ISS, female sex, and undergoing cardiovascular surgery were associated with increased odds of mortality, whereas higher SBP, higher GCS scores, and the “Struck By/Against” mechanism of injury were associated with decreased odds of mortality. These findings are consistent with those of previous studies that have identified age, injury severity, and physiological parameters as important predictors of mortality in trauma patients [33,34,35,36]. Patients who died had a shorter total time to procedure, total length of stay, and total ICU length of stay than those who survived [29,37,38,39]. This finding may reflect the severity of their condition and the need for immediate interventions [40,41].

The present study had several limitations that should be considered when interpreting the results. The retrospective nature of the study and the use of a large national database may have introduced potential biases and limitations in data quality and completeness. Additionally, the study did not account for potential confounding factors such as the timing of tracheostomy, the specific indications for tracheostomy, and the patient’s pre-existing comorbidities, which could influence the outcomes and mortality risk. Future prospective studies with more detailed data on the timing and indications for tracheostomy and long-term outcomes could further enhance our understanding of the optimal management strategies for this patient population. Despite these limitations, this study provides valuable insights into the characteristics, outcomes, and factors associated with mortality in patients who underwent tracheostomy after traumatic injury. Further research is warranted to better understand the factors influencing the decision to perform permanent or temporary tracheostomy and the potential impact on long-term outcomes in patients with trauma. It is also important to note that tracheostomy management decisions often evolve during the course of treatment, influenced by factors such as patient recovery, complications, and changing clinical circumstances. The NTDB database does not capture the temporal progression of these clinical decisions, including the initial assessment at hospital admission, ICU course, or subsequent modifications to the airway management plan. This limitation highlights the need for prospective studies that can better document the timing and evolution of tracheostomy decision-making. The current analysis did not employ case matching, which limits our ability to draw causal conclusions about factors influencing tracheostomy outcomes. A matched-pair analysis controlling for injury characteristics, procedures, and patient factors would better elucidate whether observed differences between groups represent true associations or reflect underlying case-mix variations.

## 5. Conclusions

This study compared the demographics, clinical characteristics, outcomes, and factors associated with mortality between patients undergoing permanent and temporary tracheostomies after traumatic injury using NTDB data. Patients who required permanent tracheostomy had a higher proportion of severe injuries and a higher prevalence of cardiovascular health issues and blood disorders. Logistic regression analysis identified older age, higher ISS, female sex, and cardiovascular surgery as factors associated with increased mortality. The tracheostomy type was not significantly associated with mortality, suggesting that other factors influenced this decision. Patients who died had a shorter time to procedure, length of stay, and ICU stay and a higher sum and proportion of complications. These findings highlight the importance of considering patient factors when assessing mortality risk and making clinical decisions regarding tracheostomy in patients with trauma. Close monitoring and management of complications are crucial in this patient population. Further prospective studies are warranted to better understand the optimal management strategies for patients undergoing tracheostomies after traumatic injuries.

## Figures and Tables

**Table 1:** Comparison of Demographics and Clinical Characteristics by Tracheostomy Status.

Characteristics	Permanent Tracheostomy (*N* = 3170)	Temporary Tracheostomy (*N* = 38,460)	*p*-Value
Age	48.0 (29.0, 63.0)	47.0 (29.0, 62.0)	0.763
Age ≥ 65	716 (22.6%)	8336 (21.7%)	0.231
Sex (Female)	726 (22.9%)	9139 (23.8%)	0.548
Race			0.045
Black	548 (17.3%)	6140 (16.0%)	
Others	470 (14.8%)	6196 (16.1%)	
White	2152 (67.9%)	26,124 (67.9%)	
Mechanism			0.430
Cut/Pierce	51 (1.6%)	631 (1.6%)	
Fall	741 (23.4%)	8499 (22.1%)	
Firearm	305 (9.6%)	3740 (9.7%)	
MVT	1503 (47.4%)	18,616 (48.4%)	
Other	375 (11.8%)	4386 (11.4%)	
Intent			0.364
Assault	312 (10.1%)	4090 (10.8%)	
Other	14 (0.5%)	106 (0.3%)	
Self-inflicted	141 (4.6%)	1729 (4.6%)	
Undetermined	24 (0.8%)	292 (0.8%)	
Unintentional	2595 (84.1%)	31,615 (83.6%)	
Comorbidities			
Cardiovascular health issues	148 (4.7%)	1513 (3.9%)	0.042
Major psychiatric illness or dementia	335 (10.6%)	4072 (10.6%)	0.972
Chronic health conditions	416 (13.1%)	4978 (12.9%)	0.772
Blood disorders presence	211 (6.7%)	2180 (5.7%)	0.022

Note: Data are presented as median (Q1, Q3) for continuous variables or n (%) for categorical variables. MVT stands for motor vehicle traffic. Q1 and Q3 represent the first and third quartile, respectively.

**Table 2:** Comparative Analysis of Clinical Metrics and Demographic Characteristics by Tracheostomy Type.

Clinical or Demographic Factor	Permanent Tracheostomy (*N* = 3170)	Temporary Tracheostomy (*N* = 38,460)	*p*-Value
Glasgow Coma Scale	6.0 (3.0, 14.0)	6.0 (3.0, 14.0)	0.686
SBP	130.0 (104.0, 152.0)	130.0 (108.0, 151.0)	0.403
Severely injured (ISS > 15)	2478 (78.2%)	28,812 (74.9%)	<0.001
Hemodynamically unstable (SBP < 90 mmHg)	792 (25.0%)	9912 (25.8%)	0.329
Comatose (GCS total score ≤ 8)	1800 (56.8%)	21,582 (56.1%)	0.467
Hospital type			<0.001
For profit	521 (16.4%)	4216 (11.0%)	
Non-profit	2649 (83.6%)	34,242 (89.0%)	

Data are presented as median (Q1, Q3) for continuous variables or n (%) for categorical variables.

**Table 3:** Comparison of Clinical Outcomes and Complications by Tracheostomy Type.

Clinical or Procedure Indicator	Permanent Tracheostomy (*N* = 3170)	Temporary Tracheostomy (*N* = 38,460)	*p*-Value
Total ICU length of stay	19.0 (13.0, 27.0)	18.0 (12.0, 26.0)	<0.001
Operations on the nose, mouth, and pharynx	316 (10.0%)	4709 (12.2%)	<0.001
Operations on the cardiovascular system	2353 (74.2%)	27,393 (71.2%)	<0.001
Operations on the digestive system	2346 (74.0%)	27,968 (72.7%)	0.118
Surgical complications	139 (4.4%)	1955 (5.1%)	0.084
Mortality (deceased/expired)	151 (4.8%)	1831 (4.8%)	0.995

Data are presented as median (Q1, Q3) for continuous variables or n (%) for categorical variables.

**Table 4:** Demographics and Pre-existing Conditions Associated with Mortality Outcomes.

Variable	No (*N* = 38,652)	Yes (*N* = 2978)	*p*-Value
Age median (Q1, Q3)	47.0 (29.0, 61.0)	57.0 (40.0, 70.0)	<0.001
Age ≥ 65	7988 (20.7%)	1064 (35.7%)	<0.001
Sex (Female)	9209 (23.8%)	656 (22.0%)	0.048
Race			0.237
White	26,212 (67.8%)	2064 (69.3%)	
Black	6227 (16.1%)	461 (15.5%)	
Others	6213 (16.1%)	453 (15.2%)	
Mechanism			<0.001
Cut/Pierce	631 (1.6%)	51 (1.7%)	
Fall	8421 (21.8%)	819 (27.5%)	
Firearm	3746 (9.7%)	299 (10.0%)	
MVT	18,875 (48.8%)	1244 (41.8%)	
Other	4351 (11.3%)	410 (13.8%)	
Pedal/Pedestrian	501 (1.3%)	39 (1.3%)	
Struck By/Against	1608 (4.2%)	78 (2.6%)	
Unspecified	519 (1.3%)	38 (1.3%)	
Intent			<0.001
Assault	4154 (10.9%)	248 (8.4%)	
Other	108 (0.3%)	12 (0.4%)	
Self-inflicted	1691 (4.5%)	179 (6.1%)	
Undetermined	289 (0.8%)	27 (0.9%)	
Unintentional	31,739 (83.6%)	2471 (84.1%)	
Trauma type			<0.001
Blunt	31,641 (83.3%)	2323 (79.1%)	
Burn	571 (1.5%)	149 (5.1%)	
Other/unspecified	1392 (3.7%)	115 (3.9%)	
Penetrating	4377 (11.5%)	350 (11.9%)	
Cardiovascular health issues	1436 (3.7%)	225 (7.6%)	<0.001
Chronic health conditions	4815 (12.5%)	579 (19.4%)	<0.001
Blood disorders presence	2103 (5.4%)	288 (9.7%)	<0.001
Other major health conditions	864 (2.2%)	121 (4.1%)	<0.001

Data are presented as median (Q1, Q3) for continuous variables or n (%) for categorical variables.

**Table 5:** Comparison of Clinical and Demographic Factors by Mortality Outcomes.

Factor	No (*N* = 38,652)	Yes (*N* = 2978)	*p*-Value
Glasgow Coma Scale	6.0 (3.0, 14.0)	5.0 (3.0, 14.0)	0.004
SBP	130.0 (108.0, 151.0)	124.0 (100.0, 150.0)	<0.001
Severely injured	28,988 (75.0%)	2302 (77.3%)	0.005
Hemodynamically unstable	9779 (25.3%)	925 (31.1%)	<0.001
Comatose	21,633 (56.0%)	1749 (58.7%)	0.003
Hospital type			0.994
For profit	4398 (11.4%)	339 (11.4%)	
Non-profit	34,252 (88.6%)	2639 (88.6%)	

Data are presented as median (Q1, Q3) for continuous variables or n (%) for categorical variables.

**Table 6:** Clinical Outcomes and Procedure Indicators by Mortality Status.

Clinical or Procedure Indicator	No (*N* = 38,652)	Yes (*N* = 2978)	*p*-Value
ICU length of stay Median (Q1, Q3)	18.0 (13.0, 26.0)	17.0 (9.0, 27.0)	<0.001
Tracheostomy			0.929
Permanent tracheostomy	2942 (7.6%)	228 (7.7%)	
Temporary tracheostomy	35,710 (92.4%)	2750 (92.3%)	
Operations on the nose, mouth, and pharynx	4834 (12.5%)	191 (6.4%)	<0.001
Operations on the cardiovascular system	27,425 (71.0%)	2321 (77.9%)	<0.001
Operations on the digestive system	28,392 (73.5%)	1922 (64.5%)	<0.001

Data are presented as median (Q1, Q3) for continuous variables or n (%) for categorical variables.

**Table 7:** Logistic Regression Analysis of Factors Affecting DUC.

Variable	Odds Ratio [95% CI]	*p*-Value
Age	1.018 [1.016–1.021]	<0.001
ISS	1.004 [1.002–1.007]	<0.001
SBP	0.999 [0.998–1.000]	0.021
GCS	0.987 [0.979–0.994]	<0.001
Sex (Female)	1.187 [1.078–1.308]	0.001
Permanent tracheostomy	1.004 [0.864–1.167]	0.958
Cardiovascular surgery	1.487 [1.350–1.638]	<0.001
Mechanism (Ref: Cut/Pierce)		
Fall	0.888 [0.642–1.229]	0.475
Firearm	1.114 [0.795–1.560]	0.530
MVT	0.758 [0.551–1.043]	0.089
Other	1.158 [0.831–1.613]	0.387
Pedal/Pedestrian	0.894 [0.567–1.409]	0.629
Struck By/Against	0.571 [0.384–0.849]	0.006

Reference categories were used for categorical variables to determine the relative risk compared to the baseline group.

## Data Availability

The raw data supporting the conclusions of this article will be made available by the authors on request.

## Abbreviations

NTDB	National Trauma Data Bank
ICD-9-CM	International Classification of Diseases, 9th Revision, Clinical Modification
ISS	Injury Severity Score
GCS	Glasgow Coma Scale
SBP	Systolic Blood Pressure
LOS	Length of Stay
ICU	Intensive Care Unit
IQR	Interquartile Range
OR	Odds Ratio
CI	Confidence Interval
ROC	Receiver Operating Characteristic
IRB	Institutional Review Board

## References

[1] Xu K., De Ravin E., Fritz C., Parhar H.S., Moreira A., Rajasekaran K. (2023). Epidemiology and management of adult laryngeal trauma: An analysis of the national trauma data bank. ORL J. Otorhinolaryngol. Relat. Spec..

[2] Nahar T., Roberts A., Brigode W., Siddiqi M., Capron G., Starr F., Bokhari F. (2022). “HYPER-EARLY” Tracheostomy within 48 hours has less Complications and Better Prognosis Compared to Traditional Tracheostomy. Am. Surg..

[3] Huang H., Li Y., Ariani F., Chen X., Lin J. (2014). Timing of tracheostomy in critically ill patients: A meta-analysis. PLoS ONE.

[4] Zaponi R.D., Osaku E.F., Abentroth L.R., Marques da Silva M.M., Jaskowiak J.L., Ogasawara S.M., Leite M.A., de Macedo Costa C.R., Porto I.R., Jorge A.C. (2019). The impact of tracheostomy timing on the duration and complications of mechanical ventilation. Curr. Respir. Med. Rev..

[5] Noorbakhsh M.R., Kriley I.R. (2018). Management of severe respiratory failure in complex trauma patients. J. Emerg. Crit. Care Med..

[6] Humble S.S., Wilson L.D., McKenna J.W., Leath T.C., Song Y., Davidson M.A., Ehrenfeld J.M., Guillamondegui O.D., Pandharipande P.P., Patel M.B. (2016). Tracheostomy risk factors and outcomes after severe traumatic brain injury. Brain Inj..

[7] Spito A., Cavaliere B. (2019). A Therapeutic Education Program for patients that underwent at temporary tracheotomy and total laryngectomy: Leading to improved the “Diagnostic, Therapeutic and Assistance Path”. Acta Biomed..

[8] Muller R.G., Mamidala M.P., Smith S.H., Smith A., Sheyn A. (2019). Incidence, Epidemiology, and Outcomes of Pediatric Tracheostomy in the United States from 2000 to 2012. Otolaryngol. Head Neck Surg..

[9] Azimzadeh J.B., Sidell D.R., Balakrishnan K., Mathew R., Asija R., Rutter M.J., Meister K.D. (2024). Use of temporary tracheostomy occlusion to reduce the risk of sternal wound infection after sternotomy in congenital cardiac surgery. Cardiol. Young.

[10] Janssen J.N., Gradner G.M., Liehmann L. (2024). Long-term outcome of permanent tracheostomy management in two brachycephalic dogs using a commercial and a three-dimensional-printed silicone stent. Vet. Surg..

[11] Schibilsky D., Driessen A., White W.J., Lefering R., Paffrath T., Bouillon B., Walker T., Schlensak C., Mutschler M. (2020). Traumatic tracheobronchial injuries: Incidence and outcome of 136.389 patients derived from the DGU traumaregister. Sci. Rep..

[12] Araujo de França S., MTavares W., SPaiva W., JTeixeira M., Idris Z. (2021). Benefits of early tracheostomy in TBI patients. Advancement and New Understanding in Brain Injury.

[13] Timbrell D., Jankowski S. (2018). Management of and indications for tracheostomy in care of the critically ill patient. Surgery.

[14] Killien E.Y., Grassia K.L., Butler E.K., Mooney S.J., Watson R.S., Vavilala M.S., Rivara F.P. (2023). Variation in tracheostomy placement and outcomes following pediatric trauma among adult, pediatric, and combined trauma centers. J. Trauma Acute Care Surg..

[15] Balas M., Jaja B.N., Harrington E.M., Jack A.S., Hofereiter J., Malhotra A.K., Jaffe R.H., He Y., Byrne J.P., Wilson J.R. (2023). Earlier Tracheostomy Reduces Complications in Complete Cervical Spinal Cord Injury in Real-World Practice: Analysis of a Multicenter Cohort of 2001 Patients. Neurosurgery.

[16] Ismail M.I., Idris Z., Abdullah J.M., Rahman N.A.A., Nordin M. (2021). Comparing the outcomes of early and late tracheostomy in severe traumatic brain injury patient. Malays. J. Med. Sci..

[17] Bläsius F.M., Wutzler S., Störmann P., Lustenberger T., Frink M., Maegele M., Weuster M., Bayer J., Horst K., Caspers M. (2023). Predicting tracheostomy in multiple injured patients with severe thoracic injury (AIS ≥ 3) with the new T3P-Score: A multivariable regression prediction analysis. Sci. Rep..

[18] Yu W.-K., Chen Y.-C., Chen W.-C., Yi-Fong Su V., Yang K.-Y., Kou Y.R. (2022). Influencing factors for tracheostomy in patients with acute traumatic C3-C5 spinal cord injury and acute respiratory failure. J. Chin. Med. Assoc..

[19] Del Toro-Diez E., Ríos De Choudens C.S., Lajud S.A., Pascual-Marrero J., Baez-Bermejo A. (2023). Tracheostomy outcomes on trauma patients. OTO Open.

[20] Fokin A., Wycech J., Chin Shue K., Stalder R., Lozada J., Puente I. (2021). Tracheostomy in trauma patients with rib fractures. Eur. J. Trauma Emerg. Surg..

[21] Cinotti R., Voicu S., Jaber S., Chousterman B., Paugam-Burtz C., Oueslati H., Damoisel C., Caillard A., Roquilly A., Feuillet F. (2019). Tracheostomy and long-term mortality in ICU patients undergoing prolonged mechanical ventilation. PLoS ONE.

[22] Pal P., Sood A.S., Singla S. (2017). Early complications of tracheostomy: A study on 100 patients at a single tertiary care centre. Int. J. Otorhinolaryngol. Head Neck Surg..

[23] Eliachar I., Zohar S., Golz A., Joachims H.Z., Goldsher M. (1984). Permanent tracheostomy. Head Neck Surg..

[24] Kuwabara Y., Yamakawa K., Okui S., Miyazaki E., Uezono S. (2022). Association between surgical tracheostomy and chronic tracheal stenosis: A retrospective, single-center study. Front Med..

[25] Cheung N.H., Napolitano L.M. (2014). Tracheostomy: Epidemiology, indications, timing, technique, and outcomes. Respir. Care.

[26] Casamento A.J., Bebee B., Glassford N.J., Bellomo R. (2018). Prediction of tracheostomy in critically ill trauma patients: A systematic review. Crit. Care Resusc..

[27] Kojicic M., Li G., Ahmed A., Thakur L., Trillo-Alvarez C., Cartin-Ceba R., Gay P.C., Gajic O. (2011). Long-term survival in patients with tracheostomy and prolonged mechanical ventilation in Olmsted County, Minnesota. Respir. Care.

[28] Premraj L., Camarda C., White N., Godoy D.A., Cuthbertson B.H., Rocco P.R., Pelosi P., Robba C., Suarez J.I., Cho S.M. (2023). Tracheostomy timing and outcome in critically ill patients with stroke: A meta-analysis and meta-regression. Crit. Care.

[29] Morakami F.K., Mezzaroba A.L., Larangeira A.S., Queiroz Cardoso L.T., Marçal Camillo C.A., Carvalho Grion C.M. (2023). Early tracheostomy may reduce the length of hospital stay. Crit. Care Res. Pract..

[30] Lamba H.K., Hart L.D., Zhang Q., Loera J.M., Civitello A.B., Nair A.P., Senussi M.H., Loor G., Liao K.K., Shafii A.E. (2023). Clinical predictors and outcomes after left ventricular assist device implantation and tracheostomy. Tex. Heart Inst. J..

[31] Mastropietro C.W., Sassalos P., Riley C.M., Piggott K., Allen K.Y., Prentice E., Safa R., Buckley J.R., Werho D.K., Wakeham M. (2024). Clinical Outcomes After Tracheostomy in Children With Single Ventricle Physiology: Collaborative Research From the Pediatric Cardiac Intensive Care Society Multicenter Cohort, 2010–2021. Pediatr Crit Care Med..

[32] Jang H., Yoo W., Seong H., Kim S., Kim S.H., Jo E.J., Eom J.S., Lee K. (2024). Development of a Prognostic Scoring System for Tracheostomized Patients Requiring Prolonged Ventilator Care: A Ten-Year Experience in a University-Affiliated Tertiary Hospital. Medicina.

[33] Eghzawi A., Alsabbah A., Gharaibeh S., Alwan I., Gharaibeh A., Goyal A.V. (2024). Mortality Predictors for Adult Patients with Mild-to-Moderate Traumatic Brain Injury: A Literature Review. Neurol. Int..

[34] Cassinat J., Nygaard J., Hoggard C., Hoffmann M. (2024). Predictors of mortality and rehabilitation location in adults with prolonged coma following traumatic brain injury. PM&R.

[35] Gross M.G., Filiberto D.M., Lehrman B.H., Lenart E.K., Easterday T.S., Kerwin A.J., Byerly S.E. (2024). Outcomes and predictors of delayed intervention after renal trauma. Am. Surg.

[36] Gendler S., Gelikas S., Talmy T., Nadler R., Tsur A.M., Radomislensky I., Bodas M., Glassberg E., Almog O., Benov A. (2024). Predictors of Short-Term Trauma Laparotomy Outcomes in an Integrated Military-Civilian Health System: A 23-Year Retrospective Cohort Study. J. Clin. Med..

[37] Hochu G., Soule S., Lenart E., Howley I.W., Filiberto D., Byerly S. (2024). Synchronous tracheostomy and gastrostomy placement results in shorter length of stay in traumatic brain injury patients. Am. J. Surg..

[38] Albayrak T., Yanal H., Sengul D., Sengul I., Albayrak M., Eyüpoğlu S., Muhtaroğlu A., Cinar E. (2023). First management of percutaneous dilatational tracheostomy in severe acute respiratory syndrome coronavirus 2 akin to the vital head and neck region and thyroid gland bed: Trust, but be careful whom (you trust)?. Rev. Assoc. Med. Bras..

[39] Albert G.P., McHugh D.C., Hwang D.Y., Creutzfeldt C.J., Holloway R.G., George B.P. (2023). National Cost Estimates of Invasive Mechanical Ventilation and Tracheostomy in Acute Stroke, 2008–2017. Stroke.

[40] Charland N., Chervu N., Mallick S., Le N., Curry J., Vadlakonda A., Benharash P. (2024). Impact of early tracheostomy after lung transplantation: A national analysis. Ann. Thorac. Surg..

[41] Chinta S., Haleem A., Sibala D.R., Kumar K.D., Pendyala N., Aftab O.M., Choudhry H.S., Hegazin M., Eloy J.A. (2024). Association between modified frailty index and postoperative outcomes of tracheostomies. Otolaryngol. Head Neck Surg..

